# Application of Transcranial Direct Current Stimulation in Neurorehabilitation: The Modulatory Effect of Sleep

**DOI:** 10.3389/fneur.2016.00054

**Published:** 2016-04-06

**Authors:** James K. Ebajemito, Leonardo Furlan, Christoph Nissen, Annette Sterr

**Affiliations:** ^1^School of Psychology, Faculty of Health and Medical Sciences, University of Surrey, Guildford, UK; ^2^Department of Psychiatry and Psychotherapy, University of Freiburg Medical Center, Freiburg, Germany; ^3^Department of Neurology, University of São Paulo, São Paulo, Brazil

**Keywords:** stroke, neuromodulation, memory, motor learning, recovery

## Abstract

The relationship between sleep disorders and neurological disorders is often reciprocal, such that sleep disorders are worsened by neurological symptoms and that neurological disorders are aggravated by poor sleep. Animal and human studies further suggest that sleep disruption not only worsens single neurological symptoms but may also lead to long-term negative outcomes. This suggests that sleep may play a fundamental role in neurorehabilitation and recovery. We further propose that sleep may not only alter the efficacy of behavioral treatments but also plasticity-enhancing adjunctive neurostimulation methods, such as transcranial direct current stimulation (tDCS). At present, sleep receives little attention in the fields of neurorehabilitation and neurostimulation. In this review, we draw together the strands of evidence from both fields of research to highlight the proposition that sleep is an important parameter to consider in the application of tDCS as a primary or adjunct rehabilitation intervention.

## Introduction

Sleep disorders are often comorbid to neurological disorders, such as Parkinson’s disease (1), multiple sclerosis (2), traumatic brain injury (3, 4), and stroke (5). Their manifestation can be a direct consequence of the neuropathology, resulting from a dysfunction of neural networks that are implicated in sleep regulation. In addition, sleep disorders can emerge more indirectly mediated, for example, by medication, stress, depression, fatigue, or pain.

The onset and presentation of sleep disorders may vary widely between individuals and across different neurological disorders. For example, 74–98% of patients with Parkinson’s disease will develop sleep problems, such as a decrease in rapid eye movement (REM) sleep and total sleep time, as late as 10 years after their diagnosis (6). In stroke, more than half of patients have sleep-disordered breathing (SDB), and around 20–40% experience sleep–wake disorders, such as insomnia or excessive daytime sleepiness, at some point after their vascular insult (5). Stroke patients in the chronic phase of recovery further have poorer sleep efficiency (7) and also show greater prominence of slow-wave EEG at wake than age-matched controls (8). Moreover, explorative qualitative data revealed that patients with chronic low-functioning hemiparesis feel their sleep has deteriorated gravely since suffering the stroke, and, critically, that their difficulty sleeping is caused by the stroke and the impact it had on their physical and mental health (unpublished data). Together, these data suggest that sleep disturbances in neurological conditions are a common yet diverse problem, which is multifactorial in origin.

The presence of sleep disorders can compromise or complicate the treatment of neurological disorders. For instance, sleep problems early after stroke might interfere with acute recovery, potentially leading to further medical complications and prolonged hospitalization, and also increase the risk for a second stroke (9). Besides, stroke patients with sleep disorders can experience persistent fatigue, mood changes, lack of motivation, and decline in cognitive functioning (9). When combined with the modulatory effects of sleep on neuroplasticity and learning mechanisms (10, 11), sleeping poorly might compromise motor recovery, long-term functional outcomes, and quality of life. Moreover, we argue that sleeping poorly is likely to promote adverse health behaviors, such as a sedentary lifestyle, which in turn may aggravate poor sleep and poor health, thereby creating a vicious circle. For example, it is entirely conceivable that limited physical activity experienced by patients in the chronic phase of stroke is a potential candidate responsible for sleep difficulties through autonomic dysregulation, metabolic changes, or altered sleep/arousal promoting activity. Understanding the modulatory effects of sleep on the efficacy of neurorehabilitative interventions, such as motor training and neuromodulation, is therefore intrinsically and reciprocally linked to daytime behavior.

In this article, we will discuss the impact of sleep and its disorders on the rehabilitation of neurological patients. Using the example of stroke, we will first review the theoretical rationale underlying the application of specific modulatory therapies that aim to augment motor recovery after stroke, such as transcranial direct current stimulation (tDCS). In the subsequent section, we make the case for a link between sleep and tDCS based on the argument that they exert their biological effects through a common mechanism, that is, the modulation of neuroplasticity processes and therefore might influence each other’s outcome. For instance, tDCS, when delivered as a stand-alone intervention, might alter specific aspects of sleep, such as enhancing slow-wave sleep (SWS), which is crucial for memory formation and consolidation (12). On the other hand, sleep characteristics might influence tDCS efficacy, whether it is delivered as a stand-alone or adjunctive intervention, for example, combined with training-based motor rehabilitation therapies. Therefore, we propose that a better appreciation of the interaction between sleep, tDCS mechanisms, and neurorehabilitation is needed to maximize therapy efficacy and improve rehabilitation outcomes in patients.

## tDCS in Stroke Rehabilitation

Stroke is a major cause of disability worldwide. Rehabilitation after stroke is essential for alleviating the associated motor impairments and disabilities in patients, and currently represents an important aspect of the global stroke challenge (13). Basic science research has made an enormous contribution toward improving neurorehabilitation. This led to new training principles for motor deficits after stroke (14), which in turn contributed to a theoretical and empirical step change in the field and improved the prospect of long-term care for patients (15). Furthermore, a better understanding of the neural mechanisms of recovery and interventions included the exploration of neuromodulatory methods, such as peripheral sensory stimulation, pharmacological intervention, cell-based therapy, and brain stimulation, such as tDCS and repetitive transcranial magnetic stimulation (rTMS) (16–18). Out of these methods, tDCS has emerged as the most promising and practical approach.

**Figure 1:**
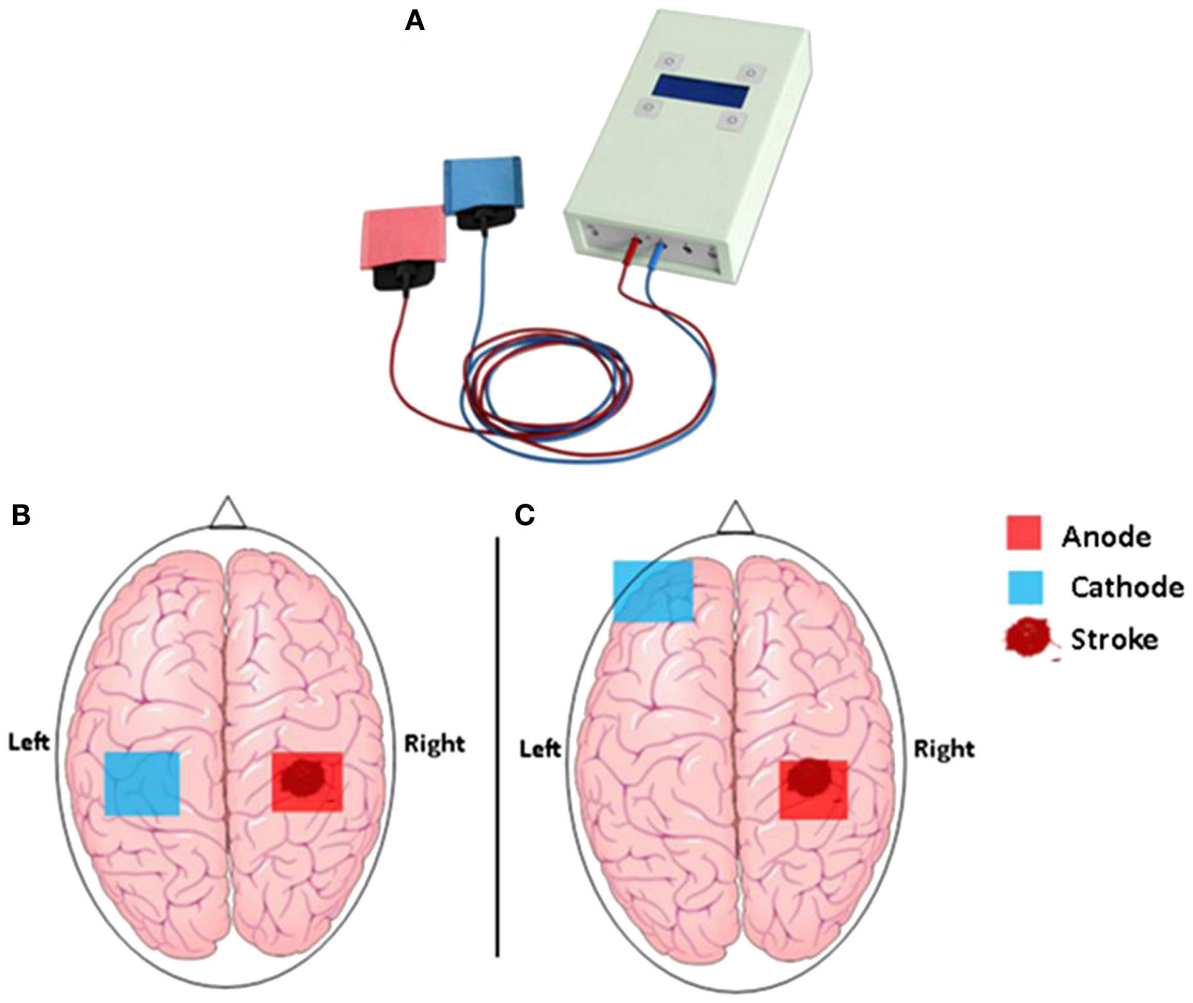
**(A)** The hand-held one-channel transcranial DC stimulator and **(B)** transcranial direct current stimulation set up to promote functional motor recovery after stroke. Following a stroke, motor deficit may occur as a result of interhemispheric inhibition from the contralesional (unaffected) M1 to the ipsilesional (affected) M1. Cathodal tDCS to the contralesional hemisphere can be used to decrease interhemispheric inhibition, while anodal tDCS to the ipsilesional M1 can be used to decrease motor deficit in the affected hemisphere. **(C)** Motor deficit after a stroke is associated with reduced participation from the ipsilesional M1. Anodal tDCS to the ipsilesional M1 can be used to enhance motor function, while cathodal tDCS to the contralateral supraorbital region is thought to be functionally ineffective.

In brief, tDCS is a safe, portable, and low-cost technique capable of altering the efficacy of brain plasticity and hence learning mechanisms. It is used to modify neuronal activity in target brain regions by delivering a weak electric current of typically 1.0–2.0 mA through two large and easily affixed surface electrodes mounted to the head (Figure 1) (19, 20). Two types of stimulation, which have opposite effects on the brain, are thereby discriminated: *anodal* tDCS, which causes a reduction in the excitability threshold of neurons (excitatory tDCS), and *cathodal* tDCS associated with a downregulation of cortical excitability (inhibitory tDCS) (21). Generally, tDCS is applied for 20–60 min, and its effect can be observed during and, importantly, for a prolonged period after the stimulation has ceased. For example, a randomized, double-blind cross-over study in stroke patients demonstrated that 20 min of 1-mA anodal stimulation over the primary motor cortex induced performance gains in the paretic hand that lasted for more than 30 min after the stimulation (22, 23). Other studies found therapeutic gains, sustained for weeks or even month, following repeated tDCS stimulation in combination with motor training over several days (24–26). Moreover, cathodal (inhibitory) tDCS has successfully been used to decrease interhemispheric inhibition from the unaffected hemisphere (27), providing an alternative pathway to recovery through increasing cortical excitability in the affected hemisphere.

The use of tDCS in stroke rehabilitation has been largely predicated on two complementary, evidence-based hypotheses. The first hypothesis assumes that motor recovery after stroke is mostly driven by neural mechanisms similar to those promoting motor learning in the intact brain, i.e., activity-dependent synaptic plasticity, characterized by both functional and structural changes in neuronal connections within spared sensorimotor circuits (28) and that such mechanisms, besides being essentially influenced by motor experience, can be further potentiated by neuromodulatory approaches (29–33). On the other hand, the second hypothesis assumes that motor deficits after stroke, such as hand paresis, often result from a complex interaction between the direct effects of the brain lesion itself, e.g., the focal disruption of motor cortex and/or its corticospinal fibres, and subsequent maladaptive plasticity processes occurring within structurally intact, residual brain circuits. The latter usually involves the combination of interacting phenomena such as depression/inactivity of perilesional motor cortex and/or corticospinal fibres and excessive transcallosal inhibition of the affected hemisphere by the opposite, unaffected hemisphere (18, 34, 35).

Based on these two key hypotheses, three types of tDCS protocols have emerged as adjunctive treatment strategies to be delivered in combination with motor training-based therapies. The first approach aims to facilitate synaptic activity and plasticity, and hence motor learning, in the ipsilesional motor cortex through excitatory anodal stimulation (17). The synaptic excitation/facilitation exerted by anodal tDCS in the affected hemisphere might contribute to improve motor function *via* at least two overlapping neural mechanisms: it may not only facilitate the recruitment of potentially spared corticospinal neurons innervating the paretic limb but also contribute to the normalization of interhemispheric imbalance by reactivating preserved, yet silent inhibitory influences from the affected motor cortex toward the contralateral motor area. The second approach consists in inhibiting/decreasing synaptic activity in the contralesional motor cortex with inhibitory cathodal stimulation (36). The inhibition of activity in the non-affected hemisphere contributes to a reduction of the increased inhibitory influence this hemisphere exerts upon the affected motor cortex. This, in turn, might contribute to the restoration of the interhemispheric equilibrium and facilitate corticospinal activation of the affected hemisphere. Finally, the third approach consists simply in combining the two in a bihemispheric stimulation protocol, with anodal stimulation of the ipsilesional and cathodal stimulation of the contralesional motor cortex provided at the same time. This combined protocol is thought to produce even greater improvements in motor function (26).

The results from studies combining tDCS with motor rehabilitation training are numerous and promising (20), highlighting the underlying potential of tDCS for improving motor outcomes beyond the levels obtained with physical practice alone. However, the evidence base required to firmly set tDCS as an effective adjunctive therapy is not yet robust (37). Many questions remain to be answered, such as the exact mechanisms by which tDCS affects neural processing that promotes motor improvements and the ideal timing between motor training and stimulation. However, the most critical question concerns the parameters needed to determine how patients can be optimally stratified according to specific markers that predict how effectively they will respond to the stimulation (20, 38). We argue that sleep is likely to be one of the parameters to be considered in this optimization.

Identifying modulators of treatment efficacy is a challenging yet important endeavor, which contributes not only to foster mechanistic understanding of the intervention itself and the targeted pathology but also, and critically, to improve methodological quality and thereby the impact of future studies testing that intervention. This is especially relevant for the process of creating the evidence base for a particular therapy. When not controlled for, those variables can critically affect the bivariate causal relationship of interest and generate misconceptions about the real efficacy of the intervention under investigation.

Within the context of motor rehabilitation, determining markers for rehabilitative therapies in patients has been a recognizable effort in the literature. For instance, Riley et al. (39) found that the degree of integrity of corticospinal fibers descending from the primary motor and dorsal premotor cortices in the affected hemisphere seems to be a better predictor of motor gains induced by robotic hand therapy when compared to baseline motor status and stroke volume measures (39). On the other hand, Sterr et al. (40, 41) recently suggested that corticospinal tract integrity may not have such an important role in mediating motor gains when considering motor therapies that address more gross upper limb functions, such as modified constraint-induced movement therapy (40, 41). Regarding neuromodulatory interventions, Ameli et al. (42) reported hand motor gains in subcortical but not in cortical stroke patients after a protocol of excitatory transcranial magnetic stimulation of the ipsilesional motor cortex (42). Similarly, Stagg et al. (43) found that anodal tDCS-induced behavioral gains in chronic stroke patients are associated with an increase in movement-related cortical activity within the stimulated ipsilesional motor cortex (43), a process that seems to be mediated by attenuation of γ-amino butyric acid (GABA) levels in that region (44). These stimulation studies suggest that both the structural – the degree of anatomical integrity – and functional – the amount of inhibitory activity within its internal circuitry – status of the targeted cortical region at baseline might be good predictors of motor outcomes after brain stimulation (43, 44).

Taken together, the studies outlined above provide insights into the mechanisms mediating treatment success in the case of specific intervention protocols, including adjuvant approaches such as tDCS. This harbors important implications for future studies investigating the involved therapies, as it may help to improve patient stratification, which in turn might contribute to the reduction of interindividual differences in outcomes and thereby maximize therapy impact (39, 44). As each stroke and the underlying recovery processes are different, the neural substrates underpinning treatment success might be preserved in some patients but not others (20, 38). Moreover, individual differences might further arise from psychological factors, such as mental health, motivation, and treatment compliance, as well as physical factors, such as fatigue, and their combined effect on sleep, all of which share a reciprocal relationship with each other as well as with the specific treatment mechanisms. These interactions can substantively influence outcome. For instance, exacerbated fatigue, particularly in cases of more severe hemiparesis, can decrease motivation and engagement with motor training, which in turn might adversely affect the neuroplasticity mechanisms driving (re)learning and recovery, and thereby limit motor gains (41, 45). Moreover, as discussed in detail below, sleep and plasticity are intrinsically linked, and poor sleep might hence have detrimental effects on stroke outcome. Because these variables are not stable across patients, i.e., individuals often present with varying degrees of decreased motivation, depression, fatigue, and sleep problems, and because they can interfere with neuroplasticity-driven recovery mechanisms, they not only might contribute to explain individual differences in rehabilitation outcomes but may also present important predictive markers for the efficacy of neurorehabilitation and neuromodulation as well as their combined application.

## Sleep as a Potential Modulator of tDCS-Based Stroke Rehabilitation

The hypothesis put forward in this article is that sleep might modulate the efficacy of plasticity-based therapies after stroke, such as tDCS, and thereby influence functional recovery during neurorehabilitation. This idea seems reasonable and justifiable by the literature. Empirical evidences have consistently reported the detrimental effect of sleep deprivation on memory, learning, and plastic processes in the brain (46–49). In addition, our hypothesis follows a long line of research suggesting a link between organic sleep disorders and a number of metabolic and cardiovascular diseases [see Ref. (50, 51) for reviews]. Whether sleep disorders modulate therapeutic interventions in such patients is presently unclear (50, 51). However, the presence of organic sleep disorders, and in particular SDB, in stroke patients is well established in the literature (9, 52); therefore, we will not cover this in detail in this review. Individuals with stroke often experience changes in sleep continuity and architecture, characterized by a reduction in total sleep time, sleep efficiency, and SWS, as well as increased sleep fragmentation and high incident rates of SDB (53, 54). The emergence of these poor sleep traits are caused by intrinsic and extrinsic factors, such as pain, fatigue, poor mental health, and immobility (intrinsic factors), as well as direct or indirect damage to the sleep regulatory pathways or prolonged hospitalization affecting regular sleep routine (extrinsic factors) (54, 55). Experimental evidence further suggests that poor sleep after stroke may worsen the condition and lead to a second stroke (9). However, the knowledge of the effect of sleep on rehabilitation is limited and, to the best of our knowledge, no study so far has addressed the role of sleep specifically in tDCS, either as an adjuvant or a stand-alone neurorehabilitation intervention. Therefore, we discuss below the candidate mechanisms for a modulatory effect of sleep on tDCS-based stroke rehabilitation and the implications for clinical practice.

### Neuroplasticity, Sleep, and tDCS

Below we discuss evidence showing an overlap between the neuroplasticity processes occurring during sleep and those occurring during motor training and tDCS stimulation. We first highlight the mechanism by which sleep and tDCS alter neural plasticity, which is critical for the formation of new neuronal connections in order to compensate for impairment after stroke. We then explore the link between tDCS and sleep mechanisms, which are relevant to motor learning and memory. Subsequently, the benefit of sleep on motor skill (re)learning, and how tDCS can be used to enhance this processes, will be put forward.

### Principles of Neuroplasticity

Neuroplasticity involves functional and/or structural modification of neuronal circuits in response to conditions of altered afferent and/or efferent demands (56). Neuronal connections are continuously remodeled throughout life, which allows the brain not only to learn new skills under healthy conditions but also to relearn previously acquired ones, for instance, to compensate for the loss of function caused by brain disease (56). The basic tenet underlying the efficacy of most of the current stroke rehabilitation therapies, including tDCS as an adjunctive or stand-alone intervention, is the modulation and potentiation of neuroplasticity processes (57). However, neuroplasticity is also a key function of sleep. Thus, sleep research conducted over the past decade or so has clearly demonstrated a pivotal role of sleep in the neuroplasticity processes subserving learning and memory consolidation (10, 11, 58–62). The exact mechanisms explaining the relationship between sleep and neuroplasticity are still under debate. However, two hypotheses offer an attractive proposition, namely, the synaptic homeostasis hypothesis and sleep-dependent synaptic formation. The synaptic homeostasis hypothesis described by Tononi and Cerelli describes sleep as an adaptive state that helps to maintain synaptic homeostasis and renormalization in order to recover from increased net synaptic strength and density that occurs during wake (63–67). Prior to sleep, synapses that are strongly activated are stabilized and consolidated, making them less prone to decay (68). In the context of motor skill learning, sleep-dependent synaptic potentiation has been shown not only to decrease decay of motor skills acquired during wake but also improve performance after subsequent sleep (69–72). This improvement in performance is thought to be due to sleep-dependent memory consolidation (11), which is the active reorganization of memory during sleep, leading to a more efficient memory storage (72, 73). In a motor skill learning task, such as the serial reaction time task (SRTT), initial memories are stored in the primary motor cortex and later transferred to the premotor and parietal cortices, where they are consolidated and stabilized during subsequent sleep (74). Positron emission tomography (PET) and regional cerebral blood flow measurements during sleep show more activity in brain regions involved in the execution of SRTT during wake (75). This may be due to the ongoing consolidation and stabilization of acquired skills during sleep.

In addition to the synaptic homeostatic hypothesis mechanism, which we propose as a potential mechanism by which sleep promotes plasticity in the motor cortex after stroke, sleep-dependent synaptic strengthening and growth may also be an advocate. Initial data from animal studies further suggest that sleep can promote synaptic reorganization and growth in the motor cortex after motor training. For example, Yang et al. (76) demonstrated the effect of sleep on the remodeling of postsynaptic dendritic spines in the primary motor cortex after motor training in mice. Using transcranial two-photon microscopy, they showed that sleep promotes branch-specific dendritic spines formation in the primary motor cortex in response to motor training during wake (76). In mice that underwent motor training, the rate of dendritic spine formation in pyramidal neurons from motor cortex was significantly higher, compared to mice that did not undergo motor training. Moreover, in mice that slept after the motor training, the rate of formation and retention of new dendritic spines in the motor cortex was significantly higher, compared to the sleep-deprived mice. On the molecular level, it has been proposed that the expression of genes related to neuroplasticity processes is one of the mechanisms by which sleep consolidates memory (77). This idea is supported by several animal studies, showing increased expression of immediate early genes, essential for synaptic plasticity, during the first few hours of sleep (78–81). Although these findings have been obtained from rodent models and are not entirely conclusive, they support the notion that sleep-dependent learning and memory enhancement does not only involve synaptic downregulation but also promotes synaptic plasticity associated with learning and memory. Taken together, the studies summarized above corroborate our hypothesis on the link between sleep, tDCS-induced neuroplasticity, and stroke recovery.

Interestingly, sleep is not only beneficial for memory consolidation and stabilization but also promotes improvement in performance by reactivation of neuronal circuits (70). Insights into the role of sleep in memory enhancement dates as far back as to the 1920s. In 1924, Jenkins and Dallenbach first reported improved performance in a verbal learning task after a period of sleep compared to wake (82). Recently, these findings have been replicated using a procedural motor sequence learning task (70, 73, 83, 84). One study, in particular, reported a 33% increase in performance and 30% decrease in error rate in a sequential motor task (84). This discovery suggests that sleep is not only beneficial for memory consolidation but also enhances previously acquired skills.

Long-term retention of motor skills after training is dependent on the neuroplastic processes, as described above. A motor skill acquired through training or practice is susceptible to interference and performance deterioration over a period of wake (10). Therefore, it is perhaps no surprise that motor skill learning studies have consistently reported performance improvements between training trials filled with sleep compared to wake (73, 84). Assuming that sleep is an active ingredient of memory consolidation, rather than a passive one simply allowing for reduced interference, we presume that good quality sleep is critical for consolidation, stabilization, and offline learning. It follows that good sleep is likely to enhance effective stroke recovery and rehabilitation, while poor sleep is likely to reduce it.

In this context, tDCS is particularly interesting since the main mechanism for augmenting neuroplasticity with tDCS, as well as the functional role of sleep in neuroplasticity, is the enhancement of synaptic potentiation processes such as long-term potentiation (LTP). During wake, active synapses undergo LTP as a result of learning and experience, while during sleep, a negative feedback response known as long-term depression (LTD) occurs, which prevents those synapses from saturation (85). Moreover, it is proposed that LTP is a relevant mechanism for stroke recovery (57). For example, anodal tDCS to the primary motor cortex, in combination with motor training, can elicit long-lasting potentiation (19), and repeated tDCS over the motor cortex is able to induce LTP-like plasticity (86). LTP takes place at glutamatergic neurones (87). Glutamate binds to its receptors in the postsynaptic terminal, that is, α-amino-3-hydroxy-5-methyl-4-isoxazolepropionic acid (AMPA) receptor and *N*-methyl-d-aspartate (NMDA) receptor. NMDA receptors are ionotropic receptors that are involved in the maintenance of neuronal plasticity and memory function. Motor skill learning and consolidation are suggested to be NMDA dependent (88). Similarly, tDCS LTP-like modulation is thought to be dependent on NMDA in order to enhance learning and memory (86). Interestingly, blocking NMDA receptors suppresses the effect of anodal and cathodal tDCS in humans and also impairs sleep in rats (86, 89, 90). These finding are in line with the idea that tDCS, sleep, memory, and learning all share a common physiological substrate, which involves NMDA-dependent LTP modulation. One might further speculate that this overlap in NMDA-dependent processes may lead to a trade-off in tDCS-based neurorehabilitation if good sleep quality is compromised.

**Figure 2:**
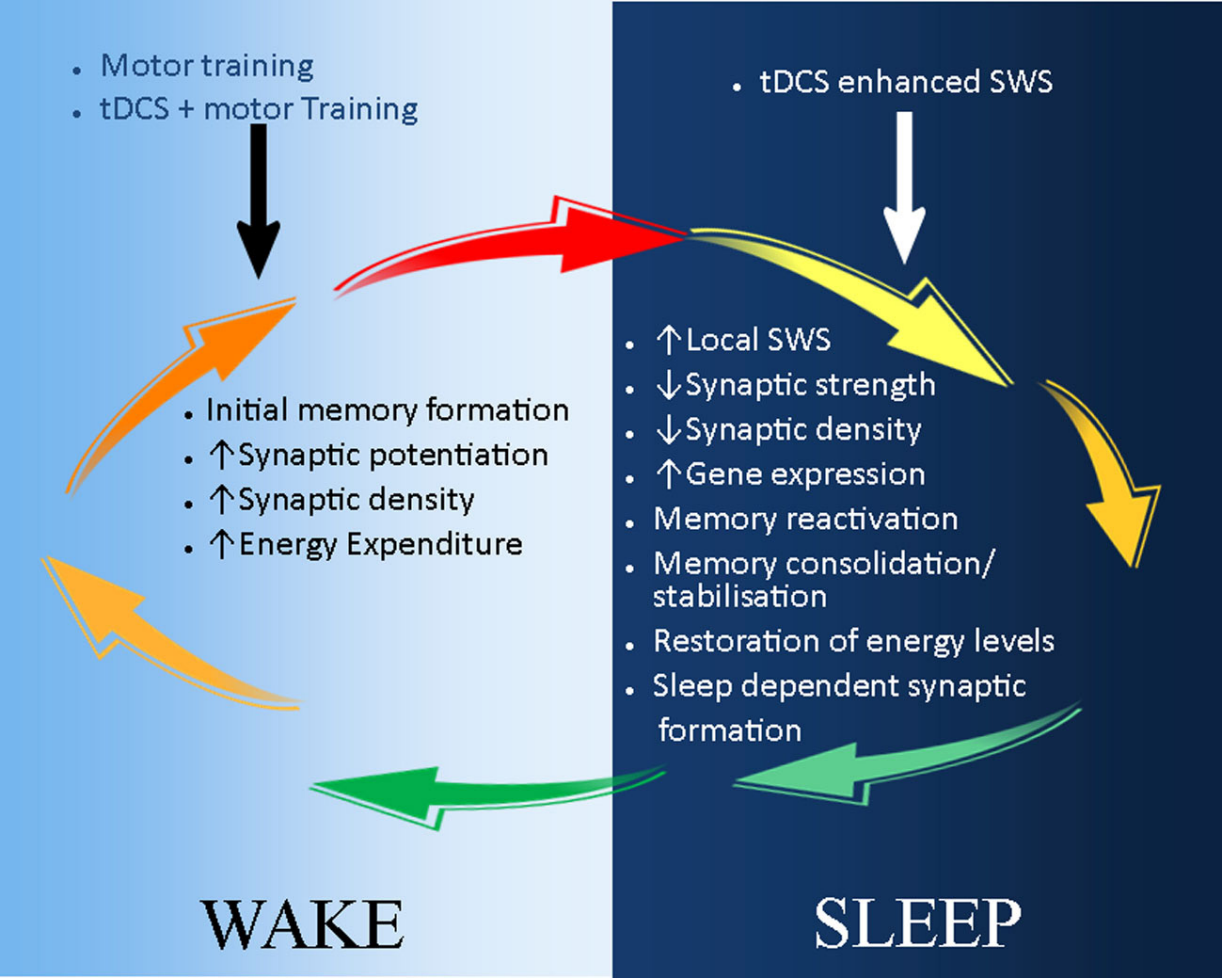
**A schematic illustration of the possible application of tDCS in stroke recovery in the sleep/wake context**. During wake (light-blue background), motor training leads to initial skill acquisition and motor memory formation. This is a result of synaptic potential and increase in synaptic strength and density. These processes can be enhanced by the combination of tDCS and motor training, which reduces the action potential threshold of neurones. During sleep (dark-blue background), synaptic downscaling occurs to restore neuronal homeostasis, and initially acquired memories are consolidated and stabilized during slow wave sleep (SWS). tDCS can be used to reduce the decay of SWS, thus further maintaining and strengthening memory consolidation and stabilization. In addition, sleep-dependent synaptic formation may also enhance tDCS-induced neuroplasticity and stroke recovery.

The most beneficial stage of sleep to learning and memory occurs during SWS (12), which is often used as an index of sleep quality because of the recovery role it plays (64, 91, 92). The modulatory effect of sleep on tDCS-based neurorehabilitation, which we emphasize in this review, most likely occurs during SWS. Studies in healthy controls have shown that consolidation of motor skills and memory formation after practice occurs during SWS (93–95). Consequently, night sleep and daytime naps rich in SWS can actively prevent memory deterioration by synaptic downscaling (73, 96). In contrast, lack of SWS has been shown to increase cognitive and memory deficit (97, 98), probably due to lack of synaptic downscaling and/or aberrant memory consolidation. Moreover, EEG studies have demonstrated that brain regions most active during wakefulness show more SWS during subsequent sleep (99–101). Studies on local sleep further demonstrate a regional increase in SWS after motor practice, specifically in those areas involved in motor control (72, 99, 102, 103). Conversely, we postulate that tDCS-enhanced motor training during wake will lead to more SWS in the brain regions involved in the execution of the task during subsequent sleep. Lastly, SWS is characterized by slow-wave oscillations (SWO), which decrease during the course of the night (67). tDCS applied during SWS reduces the decay of SWO (65, 67, 104), while anodal tDCS over frontocortical areas is beneficial to declarative memory when applied during SWS, an effect mediated by decreasing the rate of decrease of SWO (65, 105–108). Taken together, these studies identify tDCS as a stand-alone intervention to enhance SWS and boost declarative cognitive and motor memory acquired during the day over subsequent sleep. Presuming that the respective mechanisms underpinning these effects are preserved into older age, tDCS-induced enhancement of SWS might also be an effective pathway to enhance stroke rehabilitation (Figure 2).

Beyond the systems level, SWS further contributes to brain function at the cellular and local neuronal network level. In the cellular level, energy levels in terms of glycogen and ATP are restored during SWS (109, 110), whereas synaptic weight and connectivity are regulated in the neuronal level (67). This suggests that SWS may be a critical period for neuronal restoration and gaining functional recovery after stroke. Interestingly, ischemic stroke decreases SWS (111), whereas administration of SWS enhancement agent, such as γ-hydroxybutyrate (GHB), improves sensorimotor recovery following ischemic stroke (112). Therefore, this hints to two possible applications of tDCS to promote functional recovery after stroke. First, tDCS can be applied as a cognitive enhancer during motor skill learning during wake. Second, in the sleep context, tDCS can be applied during sleep to enhance SWS, thus increasing reactivation of memories and restoration of neuronal and cellular homeostasis. This proposed application takes advantage of the overlapping mechanisms of tDCS and sleep when combined in order to enhance motor recovery.

**Figure 3:**
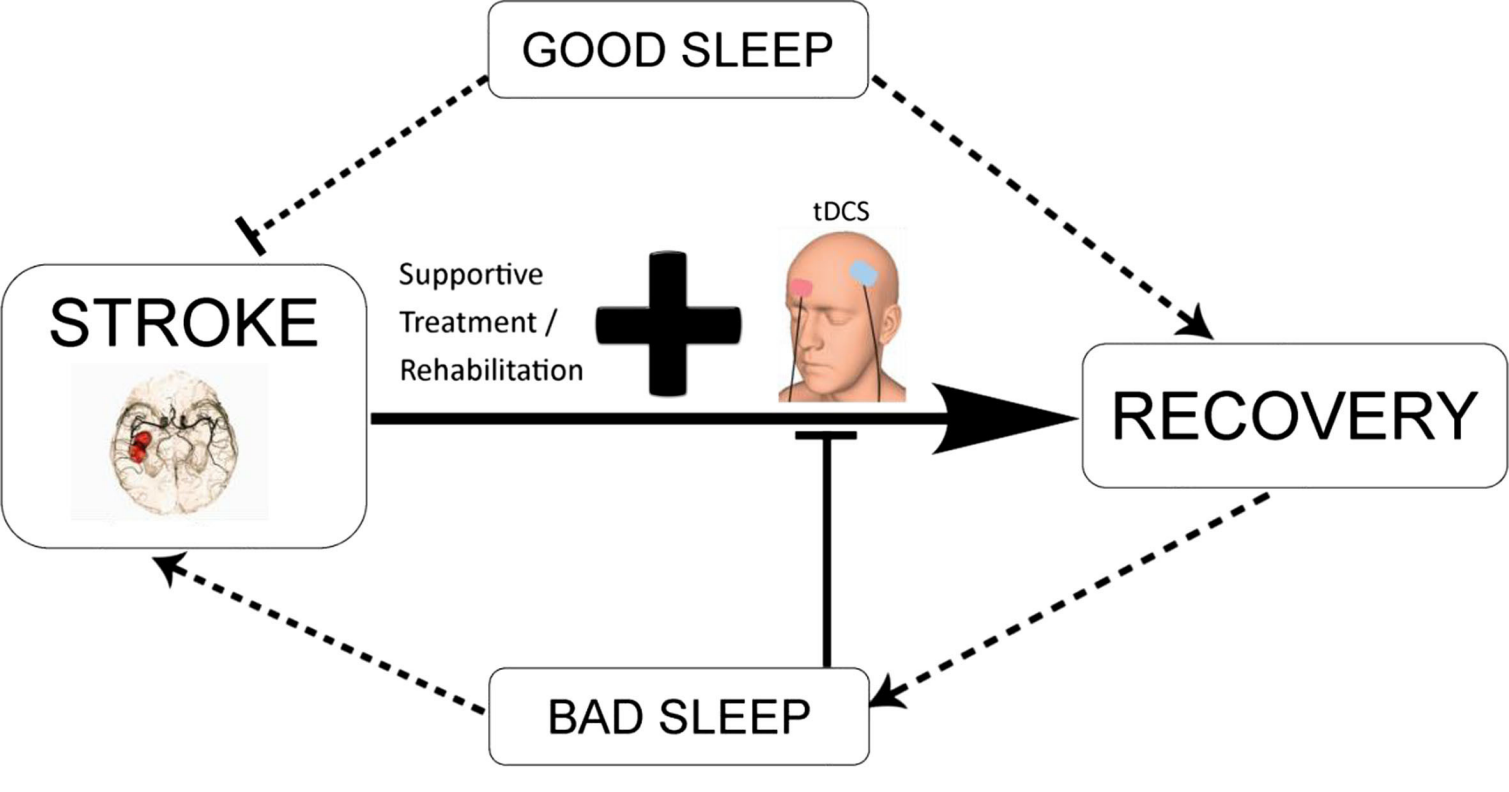
**Schematic diagram illustrating the potential interaction between tDCS-enhanced stroke rehabilitation and the modulatory effect of sleep**. tDCS in combination with conventional neurorehabilitation techniques and good sleep quality may promote functional recovery. Contrarily, bad sleep as a result of direct consequence of stroke or indirect outcome from complications associated with stroke may reduce the efficacy of tDCS-based intervention, aggravate the disease condition, and lead to long-term negative outcomes.

Taken together, good sleep habits after stroke in combination with conventional rehabilitation techniques and tDCS may promote function recovery as illustrated in Figure 3. In contrast, the evidence described in the review indicates that poor sleep after stroke may potentially lead to the occurrence of another stroke and slow down neuroplasticity processes, and hence recovery (9). Lastly, sleep my potentially modulate plasticity-based interventions, such as tDCS, by altering neuronal plasticity and other underlying mechanisms, which have not been assessed yet.

### Neurogenesis

There is much debate on the role of sleep in adult neurogenesis, although modest evidence suggests a negative effect of sleep deprivation on cell proliferation and survival (113–115). Neurogenesis is important for spatial navigation learning, long-term spatial memory retention (116, 117), as well as trace and fear conditioning (118). Available evidence suggests that sleep alone may not promote adult neurogenesis, but lack of sleep may be detrimental to the process (113, 114). For instance, glucocorticoid elevation as a result of stress arising from prolonged sleep deprivation may alter adult neurogenesis by inducing dendritic atrophy in the hippocampus (113). In relation to tDCS, Rueger et al. (119) reported that 10 days cathodal tDCS in rays increased neural stem cells proliferation. Furthermore, compelling evidence for the interaction of sleep and tDCS on neurogenesis is further coming from studies on brain-derived neurotrophic factor (BDNF) encoded by the BDNF gene. BDNF is expressed in the hippocampus and the cortex, which are the brain regions involved in learning and memory (120, 121). It is also expressed in motor neurones, where it is involved in consolidation of motor skills (120). BDNF is a neurotrophin, which promotes survival of existing neurones, as well as the growth and differentiation of new neurones and synapses. It regulates learning and memory by activating NMDA receptors through phosphorylation of one of its subunits (122). Interestingly, an association between BDNF Val(66) polymorphism with functional recovery in subcortical stroke has recently been reported (123, 124). At the same time, initial evidence also suggests that the tDCS mechanism of action, that is, NMDA-dependent LTP modulation, is driven through BDNF-dependent plasticity (120). Finally, Giese et al. (125) reported that BDNF levels are influenced by sleep, such that sleep deprivation causes a decrease in serum BDNF levels. Although this modest evidence suggests that sleep and tDCS may be beneficial to neuronal recovery by modulating neurogenesis, and possibly with involvement of BDNF, further research is warranted to investigate the molecular mechanism underlying this process.

## Conclusion

In this review, we have presented the current application of tDCS in neurorehabilitation with a focus on stroke, and also, we highlighted potential variables that can modulate tDCS-based neurorehabilitation with a focus on sleep. Furthermore, background on the concept of tDCS in brain injury treatment and the beneficial effect of good sleep and detrimental effect of poor sleep on cognitive function were provided. At present, research on the variables that can influence tDCS outcomes is relatively sparse; therefore, proposing a recommendation at this point will be rather speculative. Moreover, with the available evidence of sleep disorders prevalence and persistence in the lives of people living with neurological disorders, such as stroke, and the potential modulatory effect of sleep on tDCS efficacy, finding ways to prevent, manage, and treat sleep disorders is imperative. Furthermore, a wealth of research already suggest poor sleep is detrimental to stroke pathophysiology; therefore, it is important to establish if this relationship really exits by abolishing sleep disturbances and avoiding sleep deficit to assess if this will enhance tDCS effect in stroke patients. With this knowledge, neuropsychologists and clinicians can effectively design and implement tDCS interventions that take sleep into consideration. Lastly, a comprehensive understanding of modulators that influence the efficacy and safety of tDCS will be profitable to health professions, caregivers, citizens, and patients.

## Author Contributions

All authors listed have made substantial, direct, and intellectual contribution to the work and approved it for publication.

## Conflict of Interest Statement

The authors declare that the research was conducted in the absence of any commercial or financial relationships that could be construed as a potential conflict of interest.
